# First real-world experience with mobile health telemonitoring in adult patients with congenital heart disease

**DOI:** 10.1007/s12471-018-1201-6

**Published:** 2018-11-28

**Authors:** M. A. C. Koole, D. Kauw, M. M. Winter, D. A. J. Dohmen, I. I. Tulevski, R. de Haan, G. A. Somsen, M. P. Schijven, D. Robbers-Visser, B. J. M. Mulder, B. J. Bouma, M. J. Schuuring

**Affiliations:** 1 Department of Cardiology, Amsterdam UMC, University of Amsterdam, Amsterdam, The Netherlands; 2 Cardiology Centers of the Netherlands, Amsterdam, The Netherlands; 3 Department of Cardiology, Red Cross Hospital, Beverwijk, The Netherlands; 4 Netherlands Heart Institute, Utrecht, The Netherlands; 5 FocusCura, Driebergen-Rijsenburg, The Netherlands; 6 Department of Surgery, Amsterdam UMC, University of Amsterdam, Amsterdam, The Netherlands; 7 Department of Cardiology, Haga Teaching Hospital, The Hague, The Netherlands

**Keywords:** adult congenital heart disease, m-Health, e-Health, heart failure, arrhythmia

## Abstract

**Background:**

Arrhythmias and heart failure are common and invalidating sequelae in adult patients with congenital heart disease (CHD). Mobile health (m-Health) enables daily monitoring and a timely response that might prevent deterioration. We present an observational prospective registry to evaluate feasibility of an m‑Health telemonitoring program for managing arrhythmia, heart failure and blood pressure in symptomatic adults with CHD.

**Methods:**

Symptomatic adult patients with CHD are enrolled in an m‑Health telemonitoring program, which evaluates single-lead ECG, blood pressure and weight measurements. In case of symptoms extra measurements could be performed. Data are collected by mobile apps, matched with individualised thresholds. Patients are contacted if thresholds were exceeded or if arrhythmias were found, for treatment adjustments or reassurance. Data on emergency care utilisation, hospitalisation and patient-reported outcome measures are used to assess quality of life and self-management.

**Results:**

129 symptomatic CHD patients were invited to participate, 55 participated. Reasons for refusing consent included too time consuming to participate in research (30) and to monitor vital signs (14). At baseline 22 patients were in New York Heart Association class ≥ II heart failure, 43 patients had palpitations or documented arrhythmias, and 8 had hypertension. Mean follow-up was 3.0 months, one patient dropped out, and adherence was 97%.

**Conclusion:**

The first results indicate that this program is feasible with high adherence.

## What’s new?

- m-Health seems a very promising new tool for telemonitoring of adult patients with CHD.
- m-Health is well used by adult patients with CHD.
- m-Health is a valuable instrument to give patients immediate feedback and personalised coaching.
- m-Health results shown directly in the electronic medical records overcomes limitations mentioned in first-generation telemonitoring

## Introduction

Telemonitoring is now available and could be a powerful tool for diagnosing and treating arrhythmia and heart failure. It can also be useful for adjusting antihypertensive medication in order to reach optimal blood pressure in real-life circumstances. This may be especially true for adult patients with congenital heart disease (CHD). They are a growing patient population [1, 2]. Most of these patients need lifelong follow-up because of residual sequelae predominantly causing heart failure and arrhythmias. Contemporary care is organised by several outpatient visits per year. These visits are needed to optimise dosing of medication and detect complications or disease progression [3–7]. The current organisation of care is hampered by frequent emergency hospitalisations, possibly due to a slow response to clinical signs of deterioration.

Telemonitoring may facilitate a faster response to the first warning signs of deterioration [8]. If directly followed by properly adjusting therapy or surveillance this may result in a reduction in emergency care utilisation. Also patients may be reassured in case of benign but terrifying symptoms resulting in better patient reported outcome measurements (PROMs), quality of life and self-management[9].

Results of studies on telemonitoring in patients with heart failure are conflicting [10–13]. Several meta-analyses suggest clinical and economic benefits, but numerous prospectively initiated clinical trials have not confirmed these findings [12, 14]. Moreover, professionals are often hesitant to start using telemonitoring because they fear an overload of data with a subsequent increase in workload [15–17].

However, adult patients with CHD seem particularly suitable for telemonitoring. These patients commonly experience health-related fears and insecurities [18]. They are of a young age, they have affinity with mobile devices and a chronic condition necessitating lifelong surveillance [15, 19, 20]. In our recent study, only a small minority (14%) of adult patients with CHD were already using telemonitoring, whereas a large majority responded that they would be willing to start using it (75%) [15, 16].

An observational prospective registry was initiated to evaluate feasibility of a new mobile health (m-Health) program for telemonitoring in symptomatic adults with CHD.

## Methods

This prospective study is being conducted in a tertiary referral centre in the Netherlands. The institutional ethics committee approved the study. Informed consent is obtained in all patients. Consecutive symptomatic adult patients with CHD patients are included. Symptomatic is defined as palpitations or documented arrhythmias in the last 3 years or New York Heart Association (NYHA) heart failure class ≥ II. Patients are screened at the outpatient clinic or clinical ward and if eligible they are invited for a detailed explanation of the study. Inclusion and exclusion criteria are listed in Tab. 1. We distinguish three subgroups: patients with arrhythmias, heart failure, and hypotension or hypertension.

**Table 1:** Inclusion and exclusion criteria

*inclusion criteria*
symptomatic ACHD patients
– documented arrhythmias
– palpitations within last 3 years
– heart failure NYHA class ≥ II
age ≥18 years
possession of mobile device (e. g. smartphone, tablet)
*exclusion criteria*
impaired cognition, assessed by treating physician
tremors
asymptomatic adult CHD patients

The m‑Health program (HartWacht) consisted of hardware for single-lead ECG measurements, equipment to measure blood pressure, and a scale for body weight measurement, in combination with mobile applications to receive and transfer data. Patients were instructed on how to use the devices and mobile applications. Palpitations and arrhythmias were evaluated with single-lead ECG measurements (not only QRS complexes, but also *P* waves could be detected and visualised), which were recorded using a wireless ECG device and transferred to a remote telemonitoring centre using a smartphone application (Kardia [21]). ECGs were assessed daily by trained nurses, under supervision of a cardiologist. If an ECG was uninterpretable (artefacts) patients were asked to send a new recording. Blood pressure and weight parameters were evaluated with a blood pressure monitor (Omron) and a weight scale (i-Health) wireless connected to the patient’s smartphone. These data were transferred to the telemonitoring centre through a different smartphone application (cVitals). Data were processed using personalised thresholds and trend deviation settings, and were assessed daily. Routine measurements were done twice a week at predefined times. Patients could perform extra measurements in case of symptoms. If necessary, patients were contacted by their treating cardiologist in order to adjust therapy, for surveillance or in order to provide reassurance. Patients received an app reminder when a measurement was not performed at the required moments. Study measures (i. e. extensive history-taking and PROM questionnaires) were obtained in all participants at baseline. PROM questionnaires were repeated every 3 months. Results were automatically added to the electronic medical record (EMR) of the patient.

The following data are collected during follow-up: 1) emergency care utilisation and 2) PROMs; 3) number of visits to the outpatient clinic; 4) number of telephone contact moments; and (5) medication changes induced by results of the telemonitoring program. Additionally, we were interested in the percentage of consenting patients. Emergency care utilisation was defined as any unplanned visit to the hospital due to cardiac-related symptoms. Outpatient clinic visits were defined as a visit to a cardiologist, cardiologist in training, heart failure nurse or dedicated adult CHD nurse. An outpatient clinical visit was defined as unplanned if the electronic medical record (EMR) explicitly stated that the patient was seen pre-emptively in case of symptoms. Contact moments were defined as a contact initiated by a cardiologist, cardiologist in training or specialised nurse by telephone or email. Interventions were defined as alteration of medication, catheterisations, pacemaker or ICD implantation, electrical cardioversions, catheter-based interventions and any type of open-heart surgery. All the data were extracted from the EMR, so only events registered in the EMR were used. Historical data of care utilisation from the last year before inclusion were obtained to have an indication of disease burden of these patients.

Careful evaluation of the patient experienced health status, quality of life and self-management was performed using the PROM questionnaires. Three PROM questionnaires were used (EQ-5D-5L, PAM-13 and CaReQoL Chronic Heart Failure (CHF))[22–24]. EQ-5D-5L and CaReQoL CHF evaluate quality of life, EQ-5D-5L scores general quality of life and health status and CaReQoL CHF uses three subcategories to specify the quality of life: experienced safety, social and emotional problems and physical restrictions. PAM-13 gives an indication of the level of self-management of a patient.

For statistical analyses, SPSS 25.0 (SPSS Inc., Chicago, Illinois) for Windows was used. A two-tailed probability value of <0.05 was considered statistically significant. Descriptive data are presented as numbers with percentage, as mean with standard deviation or as median with range.

## Results

**Fig. 1:**
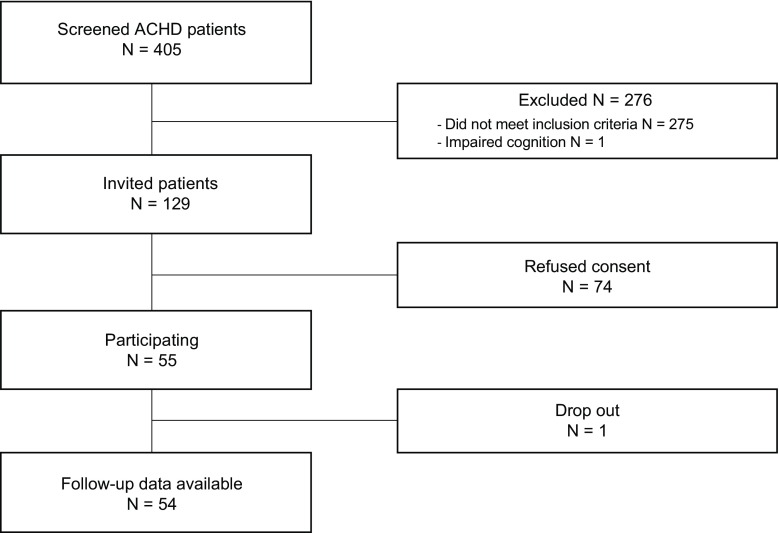
Flowchart of the study, *N* = number

Patient enrolment started in June 2017 (Fig. 1). Up to March 2018, 129 symptomatic adult CHD patients were eligible, of whom 55 (43%) consented to participate (median age of 45 years (range 19–70), 34.5% male and CHD severity of mild (*n* = 6), moderate (*n* = 29) and severe (*n* = 20)). Reasons for refusing consent are shown in Tab. 2. At baseline 22 patients were in NYHA class ≥ II heart failure, 43 patients had palpitations or documented arrhythmias and eight patients were known with hypertension. Baseline characteristics of the study population are summarised in Tab. 3.

**Table 2:** Reasons for refusing consent

reasons	number (%)
too time consuming to participate in research	30 (40.5)
too time consuming to monitor vital signs	14 (18.9)
cost of health insurance deductibles	5 (6.8)
expected decrease in quality of life	17 (23.0)
no mobile device	0 (0)
emigration	1 (1.4)
other	7 (9.5)
total	74

**Table 3:** Baseline characteristics

characteristics	total patients (*n* = 55)	heart failure (*n* = 22)	palpitations or arrhythmia (*n* = 43)	hypertension (*n* = 8)
median age (years)	45 (19 to 70)	45.5 (19 to 66)	45 (21 to 70)	60 (32 to 70)
male (%)	19 (34.5)	9 (40.9)	14 (32.6)	4 (50.0)
*severity of CHD*				
– mild (%)	6 (10.9)	0 (0)	6 (14.0)	2 (25.0)
– moderate (%)	29 (52.7)	12 (54.5)	21 (48.8)	4 (50.0)
– severe (%)	20 (36.4)	10 (45.5)	16 (37.2)	2 (25.0)
history of cardiac surgery (%)	52 (94.5)	22 (100)	40 (93.0)	8 (100)
pacemaker (%)	11 (20.0)	6 (27.3)	10 (23.3)	1 (12.5)
arrhythmia at baseline (%)	43 (78.2)	10 (45.5)	43 (100)	6 (75.0)
*NYHA class*				
– II (%)	17 (30.9)	17 (77.3)	6 (14.0)	2 (25.0)
– III (%)	5 (9.1)	5 (22.7)	4 (9.3)	1 (12.5)
– IV (%)	0 (0)	0 (0)	0 (0)	0 (0)
*symptoms*				
– palpitations (%)	31 (56.4)	5 (22.7)	31 (72.1)	5 (62.5)
– dyspnoea (%)	8 (14.5)	7 (31.8)	4 (9.3)	0 (0)
– chest pain (%)	4 (7.3)	1 (4.5)	4 (9.3)	0 (0)
– near collapse (%)	2 (3.6)	2 (9.1)	2 (4.7)	0 (0)
– dizziness (%)	7 (12.7)	4 (18.2)	7 (16.3)	1 (12.5)
– no symptoms (%)	15 (27.3)	8 (36.4)	7 (16.3)	3 (37.5)
*RV function*				
– poor (%)	3 (5.5)	1 (4.5)	3 (7.0)	0 (0)
– moderate (%)	16 (29.1)	8 (36.4)	13 (30.2)	6 (75.0)
– good (%)	36 (65.5)	13 (59.1)	27 (62.8)	2 (25.0)
*medication*				
– antiarrhythmics (%)	35 (63.6)	14 (63.6)	31 (72.1)	8 (100)
– diuretics (%)	12 (21.8)	10 (45.5)	9 (20.9)	2 (25.0)
– anticoagulation (%)	28 (50.9)	8 (36.4)	27 (62.8)	5 (62.5)

* Data are number of patients (percentage), median (range) or mean (±standard deviation)

*CHD* congenital heart disease, *NYHA* New York Heart Association, *RV* right ventricle

One patient dropped out before the first measurement because of difficulties experienced during installation of the smartphone applications and devices. Mean follow-up was 3.0 months, adherence was 97%.

**Fig. 2:**
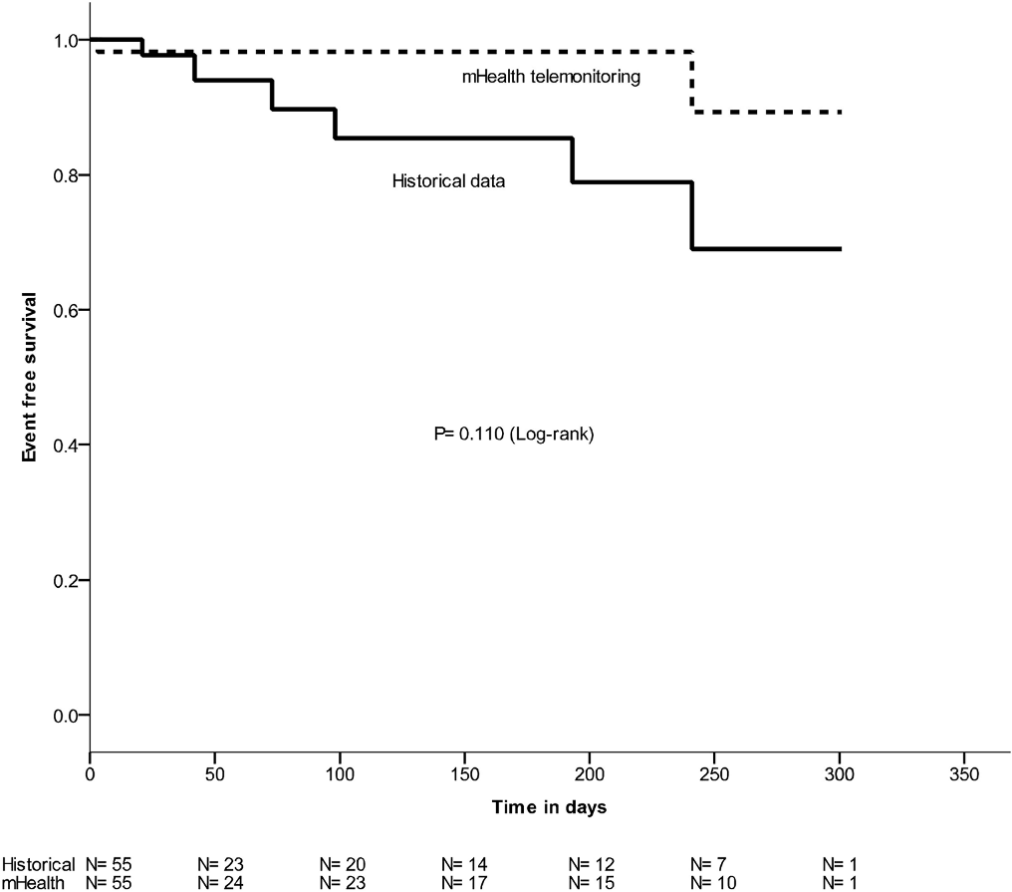
Event-free survival of patients with adult congenital heart disease (*dotted line* events during m‑Health telemonitoring, *straight line* events in historical data)

During follow-up two emergency presentations and one hospitalisation was recorded (Fig. 2). This figure also contains historical data to give further insight, demonstrating ten emergency presentations and nine hospitalisations.

**Fig. 3:**
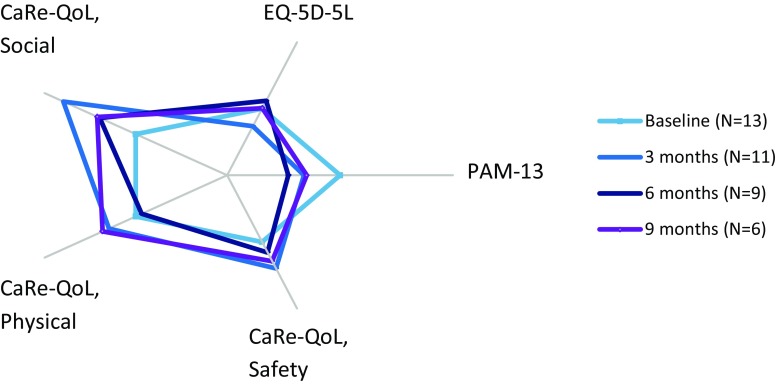
Patient-reported outcome measures (*light blue* baseline, *blue* 3 months, *dark blue* 6 months). Baseline is set as median. (*Self-management* PAM-13, *quality of life, general* EQ-5D-5L, *Safety* CaReQoL safety, *Physical* CaReQoL physical restrictions, *Social and emotional* CaReQoL social and emotional)

Serial PROM questionnaires were available for 12 patients at baseline, nine patients after 3 months and six patients after 6 months and showed a non-significant change in quality of life during m‑Health telemonitoring (Fig. 3) Compared with baseline mean scores of PROM questionnaires, quality of life (CaReQoL CHF (social, physical and safety) and EQ-5D-5L) improved by 51.7% (*p* = 0.502), 14.3% (*p* = 0.28), 3.3% (*p* = 0.87) and 0.2% (*p* = 0.89) respectively. Interestingly, patient-reported self-management decreased by 7.3% (*p* = 0.153). Also after a first increase in PROMs, this positive effect seems to fade, yet a small positive effect remains.

During follow-up 13 patients visited the outpatient clinic 19 times, medication changes were made in six patients based on telemonitoring measurements. Twelve patients had 21 telephone contacts with their cardiologist (19 were for reassurance, two were referrals to the outpatient clinic for further follow-up). One patient improved in functional class after increasing the dose of diuretics after two consecutive threshold-exceeding weight measurements. In two patients antiarrhythmic treatment was adjusted and in three patients antihypertensive treatment was adjusted.

**Fig. 4:**
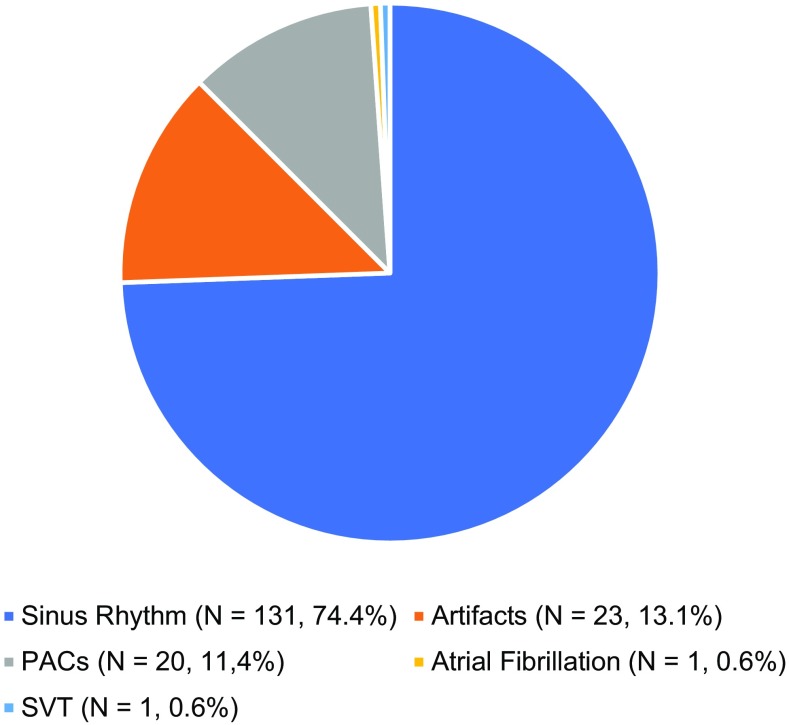
176 rhythms in 17 patients during palpitations in the first 3 months, *N* = number, %

Single-lead ECG measurements were performed frequently, and turned out to predominately be sinus rhythm. In Fig. 4 the rhythms of 176 ECGs during symptoms are shown. The majority (74.4%) of the patients could be reassured since sinus rhythm was found while the patient experienced palpitations. In one patient with palpitations, previously undiagnosed atrial fibrillation was found and another ECG also showed asymptomatic sinus node dysfunction. Larger scaled studies are warranted to distinguish which subgroup has most benefits from which type of telemonitoring.

## Discussion

The study is the first prospective study that evaluates telemonitoring through a comprehensive m‑Health program in adult patients with CHD. The program is well used by symptomatic adults with CHD. The program is feasible with a high adherence.

Data on telemonitoring in patients with heart failure are still conflicting [10–13]. Several meta-analyses suggest clinical and economic benefits, but numerous prospectively initiated clinical trials have not confirmed these findings [12, 14]. Reasons for the lack of success could be classified into six dimensions: clinical, economic, user perspective, educational, organisational, and technical [20, 25–27]. A meta-analysis of 16 high-quality randomised controlled trials showed that telemonitoring overall yields hardly any significant improvement for the average patient. However, interventions based on personalised coaching and feedback have been associated with successful results [27]. The purpose of the unique infrastructure of our m‑Health telemonitoring program (HartWacht) is to overcome limitations mentioned in these earlier studies in order to improve the benefits. This program consists of apps on patients’ mobile devices, wireless-attached to devices for measuring heart rhythm, body weight and blood pressure, controlled by a dedicated team of cardiac care nurses and cardiologists. Results are directly shown in the patient’s EMR. The treating physician could easily find an overview of measurements and therapy in the EMR and this is also true for the PROMs. This makes performing measurements relatively uncomplicated and accessible for every patient with a mobile device. It enables instant feedback to the patient. If measurements are normal they are reassured by the app and if not, as judged by a dedicated team of well-trained nurses and cardiologists, patients receive quick and reliable feedback when they experience symptoms. The feedback could be adjustment of treatment and/or reassurance. The effect of these interventions may be recognised by the patient while using the app. This could even be true for patients who are already known and being treated for arrhythmias by medication or pacemaker. If these patients suspect a deterioration of the arrhythmia they are able to take immediate action to monitor their heart rhythm and get a direct response.

The program seems to be feasible and the program was well used by our patient cohort. Our recent questionnaire study demonstrated that 75% of all patients responded that they were willing to use telemonitoring. Interestingly, only 43% of all invited patients, however, decided to participate in this program. Predominantly patients refused consent because of the time-consuming nature of participating in research. Another reason was financial, as participation would cost these patients their own health insurance deductibles. A substantial number of patients not willing to participate in this study reported that they expected a decrease of their quality of life by m‑Health monitoring. This could be due to the fact that telemonitoring is time-consuming and/or confronts patients with their illness on a more frequent basis.

We postulate that careful patient selection and a program design that not only collects a huge amount of data, but is also integrated within the EMR and supports, if necessary, immediate contact between patient and treating physician might improve healthcare for these patients.

This study has several limitations. The number of patients included so far is small and follow-up duration is still short. This is a single-centre study; more participating centres are needed. The study lacks a control group, however patients are their own controls (based on retrospective historical data). Selection bias by indication could play an important role as documented arrhythmias were one of the inclusion criteria. This could potentially imply that the patient population would be more prone to be admitted for arrhythmias in the last year before inclusion. These patients could have received appropriate antiarrhythmic drug therapy leading to a reduction in visits to the emergency department during follow-up. The adult CHD population is very diverse and the degree of disease burden varies per diagnosis and per patient [28, 29]. Therefore, the role of m‑Health telemonitoring for specific subgroups of adult CHD patients cannot yet be determined from the present data. So far we have not compared costs of the HartWacht program with standard care. Equality of clinical results with historical data versus telemonitoring can still be of interest if the costs of the program are lower compared with standard care. Moreover, ECGs taken in patients with symptoms could not always be distinguished from routinely performed ECGs. Furthermore, a larger participation rate was expected. Eligible patients refused to participate for different reasons. Lessons learned from our experience with this study, for instance on measurement intensity, could be used to improve the program and effect of these improvements will be the subject of following studies.

## Conclusion

A new m‑Health telemonitoring program evaluating arrhythmia, heart failure and blood pressure is well used by symptomatic adults with CHD. The relatively young population of adults with CHD demonstrated a high adherence. m‑Health telemonitoring might be a powerful tool for diagnosing and managing arrhythmias and heart failure. It can also be useful for adjusting antihypertensive medication in order to reach optimal blood pressure in real-life circumstances. This could result in better quality of healthcare in this patient group. However randomised control trials are needed to prove this hypothesis.
